# Biocontrol Activity of the Local Strain of *Metschnikowia pulcherrima* on Different Postharvest Pathogens

**DOI:** 10.1155/2014/397167

**Published:** 2014-04-17

**Authors:** Sezai Türkel, Mihriban Korukluoğlu, Mümine Yavuz

**Affiliations:** ^1^Department of Molecular Biology and Genetics, Faculty of Arts and Sciences, Uludag University, 16059 Bursa, Turkey; ^2^Department of Food Engineering, Faculty of Agriculture, Uludag University, 16059 Bursa, Turkey

## Abstract

The strains of the yeast *Metschnikowia pulcherrima* have strong biocontrol activity against various microorganisms. Biocontrol activity of *M. pulcherrima* largely depends on its iron immobilizing pigment pulcherrimin. Biocontrol activity of pulcherrimin producing strain, *M. pulcherrima* UMY15, isolated from local vineyards, was tested on different molds that cause food spoilage. *M. pulcherrima* UMY15 was a very effective biocontrol agent against *Penicillium roqueforti*, *P. italicum*, *P. expansum*, and *Aspergillus oryzae* in in-vitro plate tests. However, the inhibitory activity of *M. pulcherrima* UMY15 was less effective on *Fusarium sp.* and *A. niger* species in biocontrol assays. In addition, *M. pulcherrima* UMY15 strain completely inhibited the germination and mycelia growth of *A. oryzae*, *A. parasiticus*, and *Fusarium* sp. spores on artificial wounds of apples when they coinoculated with *M. pulcherrima* UMY15. Moreover, when coinoculated, *M. pulcherrima* UMY15 strain also inhibited the growth of *P. roqueforti*, *P. italicum*, *P. expansum*, *A. oryzae*, *Fusarium sp*., and *Rhizopus sp*. in grape juice, indicating that *M. pulcherrima* UMY15 can be used as a very effective biocontrol yeast against various species of postharvest pathogens, including *Penicillium*, *Aspergillus*, *Fusarium*, and *Rhizopus*.

## 1. Introduction 

Postharvest spoilage of fruits and vegetables by various molds results in substantial economic loss every year [1]. Certain species of* Aspergillus*,* Penicillium*,* Fusarium*, and* Rhizopus* are the major molds that can be found regularly on food stuff throughout the world. Some species or varieties of these molds also produce mycotoxins [2, 3]. Use of traditional fungicidal chemicals in postharvest disease control results in the formation of drug resistant microorganisms and large scale environmental pollution, which also causes severe health problems in human populations [4, 5]. Biological control of postharvest diseases caused by various fungal pathogens seems to be the best alternative to chemical fungicidal agents [6–8].

Different yeast species have been used as effective biocontrol agents against certain fungal pathogens [8–11]. Some of these yeasts are* Metschnikowia pulcherrima, Trichosporon pullulans, Rhodotorula glutinis*,* Pichia membranifaciens, *and* Pichia anomala*. Each one of these biocontrol or antagonistic yeasts can effectively inhibit growth of the various fungal pathogens on different fruits and vegetables. Some of these yeasts or their products have been commercially produced by different companies as biocontrol agents [9, 12]. Biocontrol yeasts inhibit the growth of targeted pathogens by different mechanisms. Competition for nutrient and space, secretion of specific lytic enzymes, and synthesis and secretion of specific inhibitory secondary metabolites are the only few examples of the mechanisms of the action for the biocontrol yeasts [8, 13].

Different strains of the yeast* M. pulcherrima* have been used as a highly effective biocontrol agent against different fungal species such as* Penicillium expansum* and* Botrytis cinerea* [14, 15]. Antagonistic effects of different* M. pulcherrima* strains on different species of* Candida*,* Aspergillus*,* E. coli*,* Proteus vulgaris*,* Trichosporon mucoides*, and on* Trichoderma *spp., were also reported [16].* M. pulcherrima* produces a secondary metabolite pulcherrimin and secretes it to the growth medium [17]. Pulcherrimin forms a chelate complex and immobilizes the iron ions in the growth medium [15, 18]. Hence, it seems that* M. pulcherrima* strains exert their antagonistic effects on the other microorganisms by the depletion of iron in the growth medium [15, 16, 18]. In addition to the pulcherrimin pigment, there is evidence indicating that* M. pulcherrima* also secretes lytic enzymes such as chitinase that contribute to the overall antagonistic effects of related* M. pulcherrima* strains [19].

In this study, we have tested the antagonistic effects of the recently isolated* M. pulcherrima* strain on different fungal species involved in food spoilage. We have found that the* M. pulcherrima* UMY15 strain, isolated from a local vineyard in Turkey, is a very effective biocontrol agent against different species of* Penicillium*,* Aspergillus,* and* Fusarium* on synthetic growth medium and also on apple.

## 2. Materials and Methods 

### 2.1. Microorganisms and Growth Medium

Isolation and characterization of* M. pulcherrima* UMY15 strain were described previously [16].* M. pulcherrima* UMY15 strain was cultivated in YPD medium (1% yeast extract, 2% peptone, and 2% glucose) for antagonistic activity tests.

Food spoilage molds used in this study are* P. roqueforti*,* P. italicum*,* P. expansum*,* Fusarium *sp.,* Rhizopus *sp.,* A. niger*,* A. oryzae*, and* A. parasiticus*. All of the molds are held in the culture collection of the Food Engineering Department of Uludag University (Bursa, Turkey). Molds were sporulated on malt extract agar (MEA) plates for 7 days at 30°C. Then, spores were collected aseptically into filter-sterilized 10 mL 0.1% Tween 80. The numbers of mold spores in Tween suspensions were adjusted to 10^5^ spores/mL using sterile 0.1% Tween solution [20]. Spore concentrations were determined microscopically using Thoma slides. Spore suspensions were used immediately after preparations for antagonistic activity tests.

### 2.2. Antagonistic Activity Tests

First,* M. pulcherrima* UMY15 strain was cultivated in 10 mL YPD medium overnight at 30°C in an incubator shaker with 130 rev/min at 30°C to obtain saturated precultures. Then, from these precultures, 200 *μ*L of yeast sample was inoculated into 10 mL YPD medium and grown to the midlog stage (OD_600_ : 1.0) under same growth conditions. Yeast cells were harvested by centrifugation (1500 g for 5 min) and washed once with 10 mL of sterile distilled water and resuspended in 10 mL of sterile distilled water. This* M. pulcherrima* suspensions are used in antagonistic activity tests as described below.

For antagonistic activity tests, 100 *μ*L of spore suspensions from each mold species (approximately 10^4^ spores) was taken from the stock spore suspensions and spread evenly on synthetic dextrose (SD) medium (1.67 g/L yeast nitrogen base, 5 g/L ammonium sulfate, 20 g/L glucose, and 20 g/L agar). When the surface of spore-spread plates dried, 4 *μ*L of* M. pulcherrima* UMY15 strain (prepared as described) was planted on the plates in duplicates, and then the plates were incubated at 30°C for spore germination and growth for 1-2 days. All of the antagonistic activity tests were repeated at least twice. Inhibition zones were measured manually and defined as the distance extending from the edges of the* M. pulcherrima* UMY15 colonies to the beginning of the fungal lawn on the plates and expressed in millimeters.

Antagonistic effects of* M. pulcherrima* UMY15 on germination of fungal spores were also tested when these molds were grown on apple. For this assay, apple (*Malus domestica* Borkh, cv. Golden Delicious) slices with dimensions of 4 cm L × 2 cm W × 1 cm H were prepared aseptically from the fully matured Golden Delicious apples. Then 3 holes (3-4 mm diameter, 3-4 mm deep) were prepared on these slices with sterile pipette tips. To these three holes on apple slices, 10 *μ*L of spore suspensions only, 10 *μ*L of* M. pulcherrima* UMY15 together with 10 *μ*L of spore suspensions, and 10 *μ*L of* M. pulcherrima* UMY15 samples were added, respectively. Apple slices were placed in sterile petri dishes and incubated at 25°C for 2 days for spore germination and fungal growth. At the end of incubation period, spoilage zones on the apple slices caused by the germination and the growth of molds were measured manually. Percentage of the biocontrol activity of* M. pulcherrima* UMY15 strain on the above given fungal species was expressed as the ratio of infection zones (mm) developed on apples when these molds coexist with* M. pulcherrima* UMY15 strain over the zone of infection formed by these molds alone on apple wounds [21].

Antagonistic effects of* M. pulcherrima* on the germination and hyphal growth of various molds in grape juice were also analyzed in shake-flask culture. For these assays, approximately 10^5^ of mold spores and 10^4^ colony forming unit (CFU) from* M. pulcherrima* samples were coinoculated into 20 mL of additive-free grape juice in 100 mL flask. Grape juice samples inoculated with* M. pulcherrima* and/or mold spores were incubated at 30°C in an incubator shaker with 130 rev/min. Cultures were visually inspected for mold sporulation after 48 hours. Efficacy of spore germination in grape juices was estimated by comparing the amount of mycelia growth in the grape juice cultures to the cultures that contain only spores of relevant molds (control group without* M. pulcherrima*).

The numbers given for inhibition and spoilage zones in Tables 1 and 2 are the average values of at least 4 independent experiments. The standard deviations for the zones of inhibitions were less than 10%.

## 3. Results 

### 3.1. Testing the Inhibitory Effects of* M. pulcherrima* UMY15 Strain on Fungal Growth

**Figure 1:**
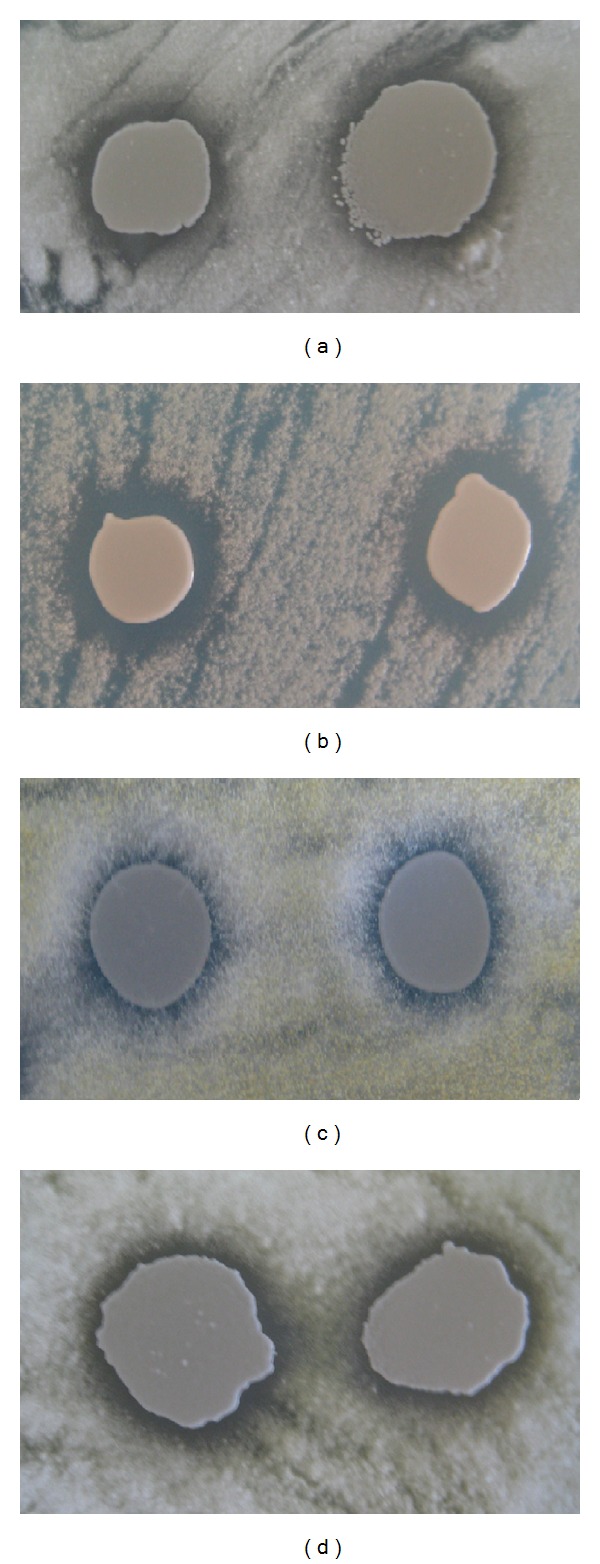
*M. pulcherrima* inhibits the growth of different molds on SD plates. Lawns on plate (a)* P. expansum*, plate (b)* P. roqueforti*, plate (c)* A. oryzae*, and plate (d)* A. parasiticus*.

The inhibitory or antagonistic effects of* M. pulcherrima* UMY15 strain on the spore germination and the growth of eight different mold species were first investigated by plate tests.* M. pulcherrima* UMY15 strain's suspensions were applied onto the spores of molds used in this study as described. Germinations and hyphal growth of* P. roqueforti*,* P. italicum*, and* P. expansum* spores were significantly inhibited by* M. pulcherrima* UMY15 strain on SD petri plates (Table 1 and Figures 1(a) and 1(b)). At the end of 24 hours incubation period, 3-4 mm of inhibition zones were clearly visible on the spore germination plates of* Penicillium* species used in this study. However, unlike* Penicillium* species, germination of* Fusarium *sp.,* Rhizopus *sp., and* A. niger* spores was inhibited at low levels by* M. pulcherrima* strain. Inhibition zones on the spore germination plates of these three fungal strains were approximately 1 mm (Table 1). Nonetheless, there is a significant level of inhibitory effect of* M. pulcherrima* strain on two other* Aspergillus* species,* A. oryzae* and* A. parasiticus, *respectively (Table 1 and Figures 1(c) and 1(d)). Inhibition zones on the spore germination plates of* A. oryzae* and* A. parasiticus* were 2-3 mm after 24 hours of incubation period.

### 3.2. Inhibition of Fungal Growth on Apple by* M. pulcherrima* UMY15 Strain

We have shown that* M. pulcherrima* UMY15 strain has a significant inhibitory effect on the spore germination and mycelial growth of certain molds such as* Penicillium* and* Aspergillus* on petri plates. Next, we wanted to test whether* M. pulcherrima* strain will also inhibit the germination of these fungal species when their spores seeded on artificial wounds on Golden Delicious apples. Hence, spore suspensions of certain species of* Penicillium*,* Fusarium*,* Rhizopus,* and* Aspergillus* were applied on small holes on apples with or without* M. pulcherrima* strain.

**Figure 2:**
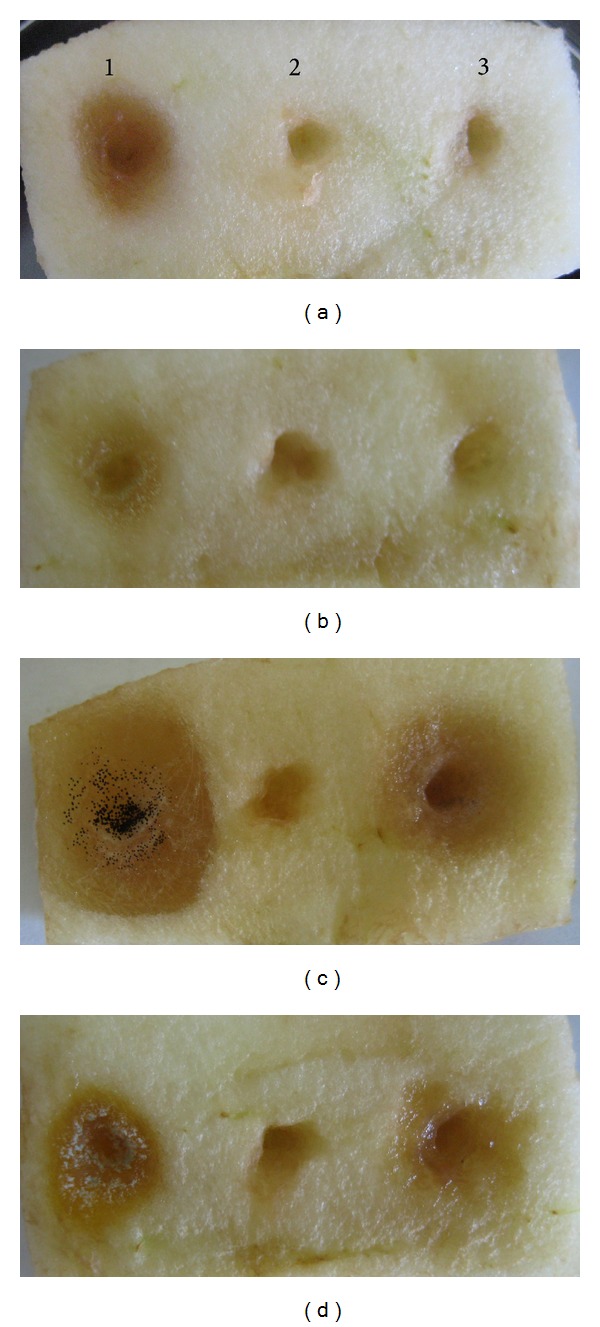
Examples of the inhibitory effects of* M. pulcherrima* UMY15 strain on different fungal species on artificially wound apples. Infection assays for* Fusarium *sp. (a),* A. Oryzae* (b),* A. niger* (c), and* A. parasiticus* (d) were done on artificial wounds on apple slices. Inoculations on artificial wounds are 1: mold spores only, 2:* M. pulcherrima* only, and 3: mold spores and* M. pulcherrima* inoculations.

Coexistence or cocultivation of* M. pulcherrima* strain UMY15 with* Fusarium *sp. spores completely inhibited the germination of* Fusarium *sp. spores and spoilage of artificially wound apples (Table 2, Figure 2(a)). In the absence of* M. pulcherrima* strain,* Fusarium *sp. spores germinated and mycelia growth led to the formation of 12 mm spoilage zones on apple wounds. However, when* M. pulcherrima* strain and* Fusarium* spores coinoculated on same wound, there were no spoilage zones on artificial wounds on apples (Table 2, Figure 2(a)). Biocontrol activity of* M. pulcherrima* on* Fusarium* was determined as 100%.

Cocultivation of* M. pulcherrima* strain with* A. oryzae* on apple wounds also prevented the spore germination, mycelia growth, and spoilage of apple slices. As seen in Figure 2(b) and Table 2, when spore suspensions of* A. oryzae* were applied on apple wounds, spore germination and mycelia growth resulted in the development of an 8–10 mm wide zone of infection. However, in the presence of* M. pulcherrima* strain together with the spores of these molds, there were no spoilage zones on apples, indicating that* M. pulcherrima* strain has very strong inhibitory effects (100% biocontrol) on the germination of the spores of these fungal pathogens.

We have also tested the inhibitory effects of* M. pulcherrima* UMY15 strain on the germination of* P. roqueforti*,* P. italicum*,* P. expansum*,* Rhizopus *sp.,* A. parasiticus,* and* A. niger*'s spores' germinations and their efficacy in the formation or development of rotting zones on artificial apple wounds. Since apple is not a natural habitat for* P. roqueforti*, there was no spore germination and fungal growth on apple holes that contain* P. roqueforti* spores at the end of a 2-day incubation period. In a similar manner, we were unable to detect any spore germination and fungal growth on apple wounds which contain* P. expansum* spores at the end of a 2-day incubation period. However,* M. pulcherrima* strain had a significant level of biocontrol activity (50–70% inhibition) on the germination and mycelia development of* P. italicum*,* A. niger,* and* A. parasiticus* (Figures 2(c) and 2(d)). Germination and the development of* P. italicum* and* A. niger* spores on apple wounds in the absence of* M. pulcherrima* strain resulted in the formation of an approximately 15 mm wide zone of infection. However, cocultivation of the spores of these molds with* M. pulcherrima* strain led to the formation of about a 5-6 mm wide zone of infection on apple wounds (Table 2, Figure 2(c)). In addition, we further tested the inhibitory effects of* M. pulcherrima* on spore germination in grape juice (Table 3). The results indicated that* M. Pulcherrima* is also very effective on* P. roqueforti*,* Fusarium sp*., and* A. oryzae*, with 100% biocontrol activity (Table 3).

## 4. Discussion 

Crop protection is the major problem in the production of fresh fruits and vegetables. Large amounts of fruits and vegetables (up to 40%) are rotten by food pathogens after harvesting from the production fields [22]. One of the commonly used methods for the postharvest protection of fresh fruits is to apply antifungal chemicals. Apart from their hazards to human health, fungal pathogens develop resistance to fungicidal chemicals [4]. Hence, biocontrol of postharvest diseases by antagonistic yeasts is the best alternative to antifungal chemicals [4, 12]. Several yeast species are currently used as biocontrol agents for postharvest preservation of fruits and vegetables [23].* M. pulcherrima* is one of the best biocontrol yeasts that are used in the prevention of the postharvest spoilage of fresh fruits [14, 24]. Biocontrol activity of* M. pulcherrima* largely depends on its pulcherrimin pigment that immobilizes free iron ions in the growth medium [18, 21].

Previously, we had isolated a new strain of* M. pulcherrima* from the local vineyards of the Düzce province of Turkey [16]. Antagonistic effects of this new* M. pulcherrima* strain on human pathogen yeasts and bacteria have been shown in that previous study [16]. Certain species of* Penicillium *sp.,* Fusarium *sp.,* Rhizopus *sp., and* Aspergillus *sp. are the major cause of spoilage of fresh fruits. In addition, certain species of* Aspergillus* also produce highly toxic aflatoxins [3]. In this study, we have analyzed the biocontrol activity of one of these new* M. pulcherrima* strains (UMY15) on aforementioned postharvest pathogens both on plate tests and also on artificial wounds on apples. Our biocontrol activity tests showed that the* M. pulcherrima* UMY15 strain has very effective antagonistic activities on* P. roqueforti*,* P. italicum*, and* P. expansum* on plate tests. However,* M. pulcherrima* UMY15 is less effective on other fungal pathogens such as* Fusarium *sp. and* Rhizopus *sp. Furthermore,* M. pulcherrima* UMY15 did not have any inhibitory effect on the growth of* A. niger* at the end of a 48 hour incubation period. Although there was an inhibitory zone on the* A. niger* lawn at the end of a 24 h incubation period,* A. niger* overcomes the antagonistic effects of* M. pulcherrima* UMY15 at the end of 48 h of growth on plates. Different fungal species transport and acquire free iron from the growth medium by different mechanisms [25]. Hence it is possible that the differential antagonistic effects of* M. pulcherrima* UMY15 on different pathogenic molds might result from the differences of iron requirements of these molds for their growth and development.

Cocultivation of* M. pulcherrima* UMY15 strain with different mold spores on artificial wounds of apples indicates that this yeast is also a very effective inhibitor of fungal spore development.* M. pulcherrima* UMY15 completely inhibited (100% biocontrol) the development of* Fusarium *sp. and* A. oryzae* spores on apple wounds (Figures 2(a) and 2(b), Table 2). It is less effective on* A. parasiticus*,* A. niger*, and* P. italicum* spore germination and mycelia development on apple. However,* M. pulcherrima* UMY15 is a better inhibitor of* A. niger* on apples than the petri tests. This indicates that* M. pulcherrima* UMY15 is a better competitor for pathogenic molds when it grows on apples. This was also shown by Janisiewicz et al. [26], that* M. pulcherrima* is a highly effective antagonistic yeast for long term storage of apples (up to 6 months). In our in-vivo analyses, we have used only one dose of* M. pulcherrima* UMY15 samples (approximately 10^3^ yeasts cells/inoculums on apple wounds). Efficacy of* M. pulcherrima* can be further improved by increasing the concentration of yeast samples applied on mold spores. In addition to our results, Oro et al. [27] reported that different isolates of* M. pulcherrima* have significant lethal effects on the non-*Saccharomyces* yeast when they are coinoculated into grape must. Sisti and Savini [28] also showed that local isolates of* M. pulcherrima* strain are highly effective as antifungal agents on human related dermatophytes. They have pointed out that strains of* M. Pulcherrima* have great potential to be used in the natural treatment of certain fungal infections.

## 5. Conclusion 

Results of this study clearly indicated that* M. pulcherrima* UMY15 strain is a very effective biocontrol agent that can be used in the prevention of postharvest diseases caused by molds. This* M. pulcherrima* strain can be developed as a commercial product for postharvest protection of fruits and vegetables from fungal pathogens.

## Figures and Tables

**Table 1:** Antagonistic effects of *M. pulcherrima* UMY15 strains on the spore germination and the mycelia growth of different fungal species.

Fungal species	Zone of inhibitions (mm)^a^	
	24 h	48 h
*P. roqueforti *	4	3
*P. italicum *	3	3
*P. expansum *	3	3
*Fusarium *sp.	1	0.5
*Rhizopus *sp.	1	1
*A. niger *	1	0
*A. oryzae *	3	2
*A. parasiticus *	2	1.5

^a^Inhibition zones were measured as the distance from the edges of *M. pulcherrima* colonies to the beginning of the fungal lawns on SD plates at the 24th and 48th hours of incubations. Standard deviations were less than 10% in zones of inhibition.

**Table 2:** Inhibition of spore germination and fungal growth on the artificial wounds on apple by *M. pulcherrima* UMY15 strain.

Fungal species	Inoculations and zones (mm) of fungal growth^a^		% Biocontrol
	Fungal spores only	Fungal spores + *M. pulcherrima *	
*P. roqueforti *	NG	NG	NA
*P. italicum *	15	5	67
*P. expansum *	NG	NG	NA
*Fusarium *sp.	12	0	100
*Rhizopus *sp.	Full growth	Full growth	0
*A. niger *	15	6	60
*A. oryzae *	8	0	100
*A. parasiticus *	10	3	70

^a^Zones of fungal growth were measured as the distance from the center of artificial wounds to the ends of fungal growths (rots) on apples at the end of 48th hours of incubations.

NG: no growth on apple wounds, NA: not applicable. Standard deviations were less than 10% in zones of inhibition.

**Table 3:** Inhibition of spore germination and fungal growth in grape juice by *M. pulcherrima* UMY15 strain.

Fungal species	% mycelial growth^a^		% Biocontrol
	Fungal spores only	Fungal spores + *M. pulcherrima *	
*P. roqueforti *	100	0	100
*P. italicum *	100	50	50
*P. expansum *	100	25	75
*Fusarium *sp.	100	0	100
*Rhizopus *sp.	100	25	75
*A. niger *	100	70	30
*A. oryzae *	100	0	100
*A. parasiticus *	100	40	60

^a^Mycelial growth in grape juice was determined visually. Spore germination and growth of mycelia in control groups that do not contain *M. pulcherrima* UMY15 were accepted as %100 growth. %biocontrol activity was estimated by comparing the mycelial growth of mold spores coinoculated with M. pulcherrima UMY15 strains to the growth of controls.

## References

[1] Moss MO (2008). Fungi, quality and safety issues in fresh fruits and vegetables. *Journal of Applied Microbiology*.

[2] Frisvad JC, Jayas DS, White NDG, Muir WE (1995). ,Mycotoxins and mycotigenic fungi. *Storage Grain Ecosystems*.

[3] Khlangwiset P, Shephard GS, Wu F (2011). Aflatoxins and growth impairment: a review. *Critical Reviews in Toxicology*.

[4] Espinel-Ingroff A (2008). Mechanisms of resistance to antifungal agents: yeasts and filamentous fungi. *Revista Iberoamericana de Micologia*.

[5] Szmedra P (1997). Banning 2,4-D and the phenoxy herbicides: potential economic impact. *Weed Science*.

[6] Saxena S, Pandey AK (2001). Microbial metabolites as eco-friendly agrochemicals for the next millennium. *Applied Microbiology and Biotechnology*.

[7] Spadaro D, Gullino ML (2004). State of the art and future prospects of the biological control of postharvest fruit diseases. *International Journal of Food Microbiology*.

[8] Droby S, Wisniewski M, Macarisin D, Wilson C (2009). Twenty years of postharvest biocontrol research: is it time for a new paradigm?. *Postharvest Biology and Technology*.

[9] Punja ZK, Utkhede RS (2003). Using fungi and yeasts to manage vegetable crop diseases. *Trends in Biotechnology*.

[10] Qin G, Tian S, Xu Y (2004). Biocontrol of postharvest diseases on sweet cherries by four antagonistic yeasts in different storage conditions. *Postharvest Biology and Technology*.

[11] Haïssam JM (2011). Pichia anomala in biocontrol for apples: 20 years of fundamental research and practical applications. *Antonie van Leeuwenhoek, International Journal of General and Molecular Microbiology*.

[12] Elmer PAG, Reglinski T (2006). Biosuppression of *Botrytis cinerea* in grapes. *Plant Pathology*.

[13] Droby S, Chalutz E, Wilson CL, Wisniewski ME (1994). Mode of action of biocontrol agents of postharvest diseases. *Control of Postharvest Diseases-Theory and Practice*.

[14] Janisiewicz WJ, Tworkoski TJ, Kurtzman CP (2001). Biocontrol potential of *Metchnikowia pulcherrima* strains against blue mold of apple. *Phytopathology*.

[15] Saravanakumar D, Ciavorella A, Spadaro D, Garibaldi A, Gullino ML (2008). *Metschnikowia pulcherrima* strain MACH1 outcompetes *Botrytis cinerea, Alternaria alternata* and *Penicillium expansum* in apples through iron depletion. *Postharvest Biology and Technology*.

[16] Türkel S, Ener B (2009). Isolation and characterization of new *Metschnikowia pulcherrima* strains as producers of the antimicrobial pigment pulcherrimin. *Zeitschrift fur Naturforschung C: Journal of Biosciences*.

[17] Kluyver AJ, Van Der Walt JP, Van Triet AJ (1953). Pulcherrimin, the pigment of *Candida pulcherrima*. *Proceedings of the National Academy of Sciences*.

[18] Sipiczki M (2006). *Metschnikowia* strains isolated from botrytized grapes antagonize fungal and bacterial growth by iron depletion. *Applied and Environmental Microbiology*.

[19] Saravanakumar D, Spadaro D, Garibaldi A, Gullino ML (2009). Detection of enzymatic activity and partial sequence of a chitinase gene in *Metschnikowia pulcherrima* strain MACH1 used as post-harvest biocontrol agent. *European Journal of Plant Pathology*.

[20] López-Malo A, Alzamora SM, Palou E (2002). *Aspergillus flavus* dose-response curves to selected natural and synthetic antimicrobials. *International Journal of Food Microbiology*.

[21] Spadaro D, Vola R, Piano S, Gullino ML (2002). Mechanisms of action and efficacy of four isolates of the yeast *Metschnikowia pulcherrima* active against postharvest pathogens on apples. *Postharvest Biology and Technology*.

[22] Kader AA (2005). Increasing food availability by reducing postharvest losses of fresh produce. *Acta Horticulturae*.

[23] Melin P, Sundh I, Håkansson S, Schnürer J (2007). Biological preservation of plant derived animal feed with antifungal microorganisms: safety and formulation aspects. *Biotechnology Letters*.

[24] Piano S, Neyrotti V, Migheli Q, Gullino ML (1997). Biocontrol capability of *Metschnikowia pulcherrima* against *Botrytis postharvest* rot of apple. *Postharvest Biology and Technology*.

[25] Howard DH (1999). Acquisition, transport, and storage of iron by pathogenic fungi. *Clinical Microbiology Reviews*.

[26] Janisiewicz WJ, Saftner RA, Conway WS, Yoder KS (2008). Control of blue mold decay of apple during commercial controlled atmosphere storage with yeast antagonists and sodium bicarbonate. *Postharvest Biology and Technology*.

[27] Oro L, Ciani M, Comitini F (2014). Antimicrobial activity of Metschnikowia pulcherrima on wine yeasts. *Journal of Applied Microbiology*.

[28] Sisti M, Savini V (2014). Antifungal properties of the human *Metschnikowia* strain IHEM 25107. *Folia Microbiologica*.

